# Loss of USP28 and SPINT2 expression promotes cancer cell survival after whole genome doubling

**DOI:** 10.1007/s13402-021-00654-5

**Published:** 2021-12-28

**Authors:** Sara Vanessa Bernhard, Katarzyna Seget-Trzensiok, Christian Kuffer, Dragomir B. Krastev, Lisa-Marie Stautmeister, Mirko Theis, Kristina Keuper, Jan-Eric Boekenkamp, Maik Kschischo, Frank Buchholz, Zuzana Storchova

**Affiliations:** 1 Molecular Genetics, TU Kaiserslautern, Paul-Ehrlich-Strasse 24, 67663 Kaiserslautern, Germany; 2 Max Planck Institute of Biochemistry, Am Klopferspitz 18, 82152 Martinsried, Germany; 3 National Center for Tumor Diseases (NCT): German Cancer Research Center (DKFZ) Heidelberg, Faculty of Medicine and University Hospital Carl Gustav Carus, Technische Universität Dresden, Helmholtz-Zentrum Dresden-Rossendorf (HZDR), Fetscherstraße 74/PF 64, 01307 Dresden, Germany; 4 Medical Systems Biology, Medical Faculty and University Hospital Carl Gustav Carus, TU Dresden, 01307 Dresden, Germany; 5 Koblenz University of Applied Sciences, Joseph-Rovan-Allee 2, Remagen, Germany; 6 German Cancer Research Center (DKFZ), Heidelberg and German Cancer Consortium (DKTK) Partner Site, Dresden, Germany

**Keywords:** Cancer, Cell cycle arrest, Multipolarity, Tetraploidy, NuMA1, SPINT2, USP28

## Abstract

**Background:**

Whole genome doubling is a frequent event during cancer evolution and shapes the cancer genome due to the occurrence of chromosomal instability. Yet, erroneously arising human tetraploid cells usually do not proliferate due to p53 activation that leads to CDKN1A expression, cell cycle arrest, senescence and/or apoptosis.

**Methods:**

To uncover the barriers that block the proliferation of tetraploids, we performed a RNAi mediated genome-wide screen in a human colorectal cancer cell line (HCT116).

**Results:**

We identified 140 genes whose depletion improved the survival of tetraploid cells and characterized in depth two of them: SPINT2 and USP28. We found that SPINT2 is a general regulator of CDKN1A transcription via histone acetylation. Using mass spectrometry and immunoprecipitation, we found that USP28 interacts with NuMA1 and affects centrosome clustering. Tetraploid cells accumulate DNA damage and loss of USP28 reduces checkpoint activation, thus facilitating their proliferation.

**Conclusions:**

Our results indicate three aspects that contribute to the survival of tetraploid cells: (i) increased mitogenic signaling and reduced expression of cell cycle inhibitors, (ii) the ability to establish functional bipolar spindles and (iii) reduced DNA damage signaling.

**Supplementary Information:**

The online version contains supplementary material available at 10.1007/s13402-021-00654-5.

## Introduction

Recent cancer genome analyses have exposed the complexity of genomic alterations during tumorigenesis. One of the frequent types of cancer-associated genomic alterations is whole-genome doubling (WGD) that arises due to defects in mitosis and cytokinesis as well as via cell‐cell fusion or chromosome endoreduplication [1–3]. Computational analysis of human exome sequences from ∼4,000 human cancers suggest that approximately 37% of all tumors have undergone at least one WGD during tumorigenesis [4–6], whereas the frequency of WGD rises to 56% in metastases [7]. A passage through tetraploidy facilitates chromosomal instability and contributes to increased tumorigenicity, metastasis, formation and drug resistance, and is associated with a reduced chance of disease-free survival [4, 6, 8–10]. Tetraploid cells also show an increased tolerance to further chromosomal abnormalities [4, 8]. However, experimental induction of tetraploidy is not well tolerated in mammalian cells, which poses a question as of how can cells survive and tolerate tetraploidy [3, 11]. Identification of pathways that limit the survival of tetraploid cells may, therefore, improve our understanding of cancerous processes and pave the way for novel cancer treatments.

**Fig. 1:**
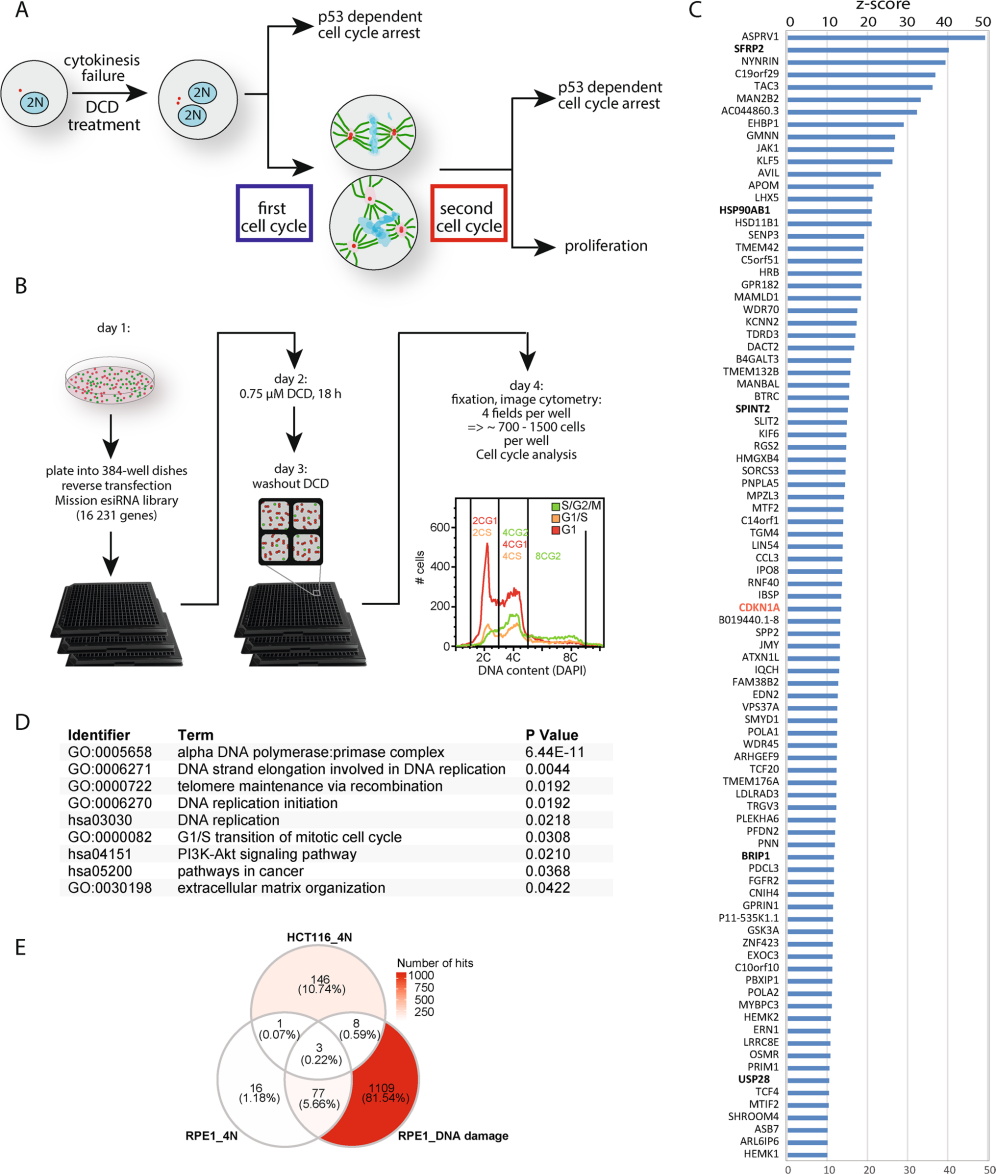
Genome-wide screen identifies novel factors involved in the proliferation of tetraploid cancer cells. **A** Schematic depiction of the possible fates of human cells upon cytokinesis failure. **B** Schematic depiction of the microscopy-based esiRNA screen set up. **C** Top candidates identified in the screen (Z-score > 10, high significance). **D** Pathway enrichment analysis identifying nine significantly enriched pathways among the screen hits. **E** Venn diagram of overlap between hits in gene depletion screens for tetraploid HCT116 cell survival improvement (HCT116_4N) and for RPE1 cell cycle arrest escape after tetraploidization (RPE1_4N) and DNA damage (RPE_DNA damage) induction [15]

*In vitro*, the proliferation of tetraploid cells arising from whole genome doubling is limited at two control points (Fig. 1A). First, cytokinesis failure induced by impairing actomyosin ring formation (e.g. by inhibition of actin polymerization with dihydrocytochalasin D (DCD), or depletion of an actin-binding protein, anilin), may trigger cell cycle arrest immediately in the following G1 phase by stabilizing the tumor suppressor protein p53 and elevating the expression of its downstream target CDKN1A [12, 13]. This is often observed in non-transformed cells, such as mouse embryonic fibroblasts (MEFs) or in the human immortalized RPE1 cell line. A genome-wide screen in RPE1 revealed that cytoskeletal stress caused by extra centrosomes in binucleated tetraploid cells activates the Hippo tumor suppressor pathway, which then via the kinases LATS1 and LATS2 inhibits the MDM2 E3 ligase, thereby stabilizing p53 [14, 15]. The G1 arrest immediately after cytokinesis failure can be bypassed by enhanced cytokine signaling [15, 16].

However, mammalian cells can often escape the G1 arrest immediately after tetraploidization and enter the cell cycle [4, 9, 17–20]. The subsequent mitoses are often aberrant due to the presence of extra centrosomes and doubled chromosome numbers (Fig. 1A). Multipolar mitosis or erroneous bipolar mitosis may also trigger a p53-dependent arrest in the subsequent G1 phase [17, 21], but little is known about the molecular mechanisms involved. Abnormal tetraploid mitosis increases oxidative damage and knockdown of ATM improves subsequent proliferation [17]. Another report demonstrated that an increased number of mature centrosomes activates the PIDDosome. This in turn leads to Caspase-2-mediated MDM2 cleavage, p53 stabilization and p21-dependent cell cycle arrest [22]. Tetraploidy may also induce replication stress and DNA damage [9, 20, 23]. Increased expression of cyclin D1 and D2 has been reported in surviving tetraploid cells, suggesting that the G1 arrest can be bypassed by enhancing the expression of G1 cyclins [24, 25]. Taken together, these findings imply that there are several molecular mechanisms that may influence the proliferation of tetraploid cells.

Meta-analysis of cancer genomes suggests that whole-genome doubling occurs not only in early precancerous lesions, but frequently also after transforming mutations of cancer driver genes, for example as an event facilitating metastasis [6, 7]. The factors that limit proliferation of transformed tetraploid cells may differ from factors important for the arrest of non-transformed ones. Therefore, we performed a RNAi-based high throughput screen for factors limiting proliferation after cytokinesis failure in the near-diploid, but transformed cell line HCT116. These cells do not arrest in the first G1 after becoming tetraploid, but enter the next cell cycle and arrest later, after subsequent aberrant tetraploid mitoses (Fig. 1A) [17]. Using siRNA and CRISPR/Cas9 techniques, we evaluated the effect of two of the identified candidates, SPINT2 and USP28. We show that SPINT2 affects mitogenic signaling as well as CDKN1A expression, and that its loss enables to bypass G1 arrest upon various cellular stresses. Additionally, we show that proliferating tetraploid cells quickly accumulate DNA damage, and that loss of USP28 reduces the checkpoint response, thus allowing proliferation of tetraploid cells. Our findings indicate that the context of tetraploidization is important for cellular response and survival, and suggest new mechanisms that enable tetraploid proliferation.

## Material and methods

### Cell lines and culture conditions

HCT116 is a human colorectal carcinoma cell line, purchased from the ATCC (No. CCL-247). HCT116-H2B-GFP cells were generated by transfection of WT cells with a pBOS-H2BGFP construct (BD Pharmingen). HCT116 FUCCI cells were generated by transfection with a plasmid encoding the N-terminus of Cdt1 fused to mKO2 (an orange fluorescent protein) which is, therefore, present only in the G1 phase (G1 sensor) and degraded in the S, G2 and M phases by the SCF complex. The G2 sensor consists of the N-terminus of Geminin fused to mAG and is, therefore, degraded between anaphase and the S phase by the APC/C complex [26]. RPE1-hTERT is a human retinal pigment epithelium cell line immortalized by telomerase expression (referred to as RPE1). RPE1 and RPE1-H2B-GFP cell lines were a gift from Prof. Erich Nigg (MPI Biochemistry, Martinsried, Germany) and Dr. Stephen Taylor (The University of Manchester, UK, Manchester Cancer Research Centre), respectively. DLD1 cells were provided by FB, whereas CaCO2 cells were a kind gift from Prof. Axel Ullrich, (MPI Biochemistry, Martinsried, Germany) and HEK293 cells were a kind gift from Prof. Stefan Kins (TU Kaiserslautern, Germany). All cells were cultured in Dulbecco´s Modified Eagle Medium GlutaMAX (DMEM) supplemented with 10% Fetal Bovine Serum (FBS) and 1% Pen-Strep (50 IU/ml penicillin and 50 μg/ml streptomycin, PAA) in a humidified cell culture incubator at 37 °C with 5% CO_2_. To passage the cells, the culture dishes were washed 2 × with PBS after which 0.25% Trypsin–EDTA was added and incubated for 3 min in the cell culture incubator. Next, cell culture medium was added to inhibit trypsin, after which the cells were collected, spun down and re-seeded.

### RNAi-based screen for factors affecting the proliferation of tetraploid cell

HCT116 FUCCI (Fluorescent Ubiquitination-based Cell Cycle Indicator) cells were reverse-transfected with an esiRNA library in 384-well black glass bottom plates on Day 1. The next day actin depolymerizing drug dihydrocytochalasin D (DCD, Sigma) was added to a final concentration of 0.75 µM, which was washed out after 18 h. 24 h later the cells were fixed with paraformaldehyde and stained with DAPI. The fluorescence intensities of the DAPI and the FUCCI signals of each nucleus were measured to establish the cell cycle profiles of cells expressing the FUCCI-G1, FUCCI-G2 or both FUCCI cell cycle probes, thereby dividing the cells into six distinguished cell cycle classes 2CG1, 2CS, 4CG2, 4CG1, 4CS and 8CG2 according to their DNA content and the FUCCI sensor expression. Cells lacking a FUCCI signal were excluded from the analysis. The relative abundance was calculated and transformed into a Z*-score value for each of the six classes. Z*-score transformation was performed for each cell cycle class by dividing the difference between its relative abundance in a particular well of a plate and the median of the whole plate by the median absolute deviation (MAD) [27]. Control wells transfected with esiRNA targeting either TP53 or KIFC1 served as positive controls. The primary screen was conducted in two technical replicates and the duplicate information was used to reduce the false negative discovery rate. A subset of 374 genes from the TP53-like category was selected for a confirmatory screen, including the identified primary TP53-like hits, and excluding genes that either were identified as hits in previous cell cycle screens [28, 29], or are located on the Y chromosome, which is not present in HCT116 cells, or were previously found to be not expressed in HCT116 cells. The confirmatory screen was performed in black 96-well glass bottom plates in four technical replicates. To confirm the primary TP53-like hits, every rescreened gene was tested against the R-LUC controls using the Dunnett's multiple comparison test; we considered a primary hit as confirmed if the *p*-value was < 0.1. Using this approach, 157 genes out of 374 primary hits were confirmed as TP53-like hits. The total confirmation rate was 42%. For more details, see Supplementary Information.

### Generation of a HCT116 H2B-GFP γ-tubulin-mRuby cell line

Human embryonic kidney cells (HEK293) were co-transfected with packaging plasmid and γ-tubulin-mRuby plasmid using lipofectamine 2000 in Optimem. The medium was replaced with culture medium 24 h post transfection. 24 h and 48 h later, medium containing viral particles was collected and filtered. Next, HCT116 H2B-GFP cells were seeded in a 6-well plate and infected using 100 µl and 600 µl supernatant per well with the addition of Polybrene. After 48 h, the cells were collected and seeded into a 15 cm cell culture dish and kept in selection medium containing Zeocin 600 μg/ml. Individual clones were screened by fluorescence microscopy and those with γ-tubulin-mRuby expression were selected for further culture.

### Formation of tetraploid cells

Cells were treated with 0.75 μM DCD for 18 h. Subsequently, the drug was washed out 3 × using prewarmed PBS, after which the cells were cultured in drug-free medium for the indicated time or immediately harvested for further experiments. Cells were seeded 24 h prior to siRNA transfection. siRNA transfection was carried out according to a standard transfection protocol using 50 nM anillin siRNA and incubated for 24 h (see transfection paragraph). Samples for flow cytometric analysis were taken 24, 30 and 48 h after anillin knockdown (KD). For microscopic analysis, the cells were fixed 24, 48 and 72 h after anillin KD.

### Generation of CRISPR Cas9 knockout cell lines

First, Cas9-containing supernatant was produced using the same protocol as for γ-tubulin-mRuby. Next, HCT116 cells were transduced with Cas9 and, 48 h later, the cells were reverse transfected with guideRNA and trackRNA in 6-well plates. Next, the transfected cells were seeded in a 15 cm dish (100 cells) to obtain single clones that were subsequently picked and tested for protein expression by immunoblotting. The guide RNAs were purchased from Dharmacon: USP28 (CR-006076–01-0002, CR-006076–02-0002, CR-006076–03-0002), SPINT2 (CR-010210–01-0002, CR-010210–03-0002, CR-010210–04-0002) and tracking RNA (U-002000–50). The arising mutations were validated by sequencing of the clones. Successful knockouts were validated via immunoblotting (Fig. S1D, E) and, in the case of SPINT2, reduction of CDKN1A (p21) expression was also used as a readout for knockout.

### Transfection assays

For reverse siRNA transfection, a transfection mix (50 nM siRNA or a mix of two different siRNAs with 50 nM each) mixed with lipofectamine 2000 and Optimem medium was prepared and pipetted into 6-well plates. Next, cells were seeded into the plates in DMEM without antibiotics and incubated for 24 h. Subsequently, the transfection medium was replaced with standard cell culture medium. For plasmid transfections, the cells were seeded in a 6-well plate and transfected using a forward protocol. A transfection mix consisting of titrated plasmid, lipofectamine 2000 and Optimem was added to the cells kept in DMEM without antibiotics. After 24 h incubation, the transfection medium was replaced with cell culture medium.

### Proliferation assay

24 h after siRNA transfection, the cells were treated with 0.75 μM DCD for 18 h. After the drug was washed out, the cells were cultured in standard conditions for the indicated time periods (0 h, 4 h, 8 h, 24 h, 30 h, 48 h). Next, the cells were pulse-labeled with 5-ethynyl-2'deoxyuridine (EdU) for 30 min, harvested and subjected to flow cytometric analysis.

### HDAC inhibition

Cells were seeded in 6-well plates and treated with 0.3 μM trichostatin A (TSA), a potent HDAC inhibitor, for 6 h and, subsequently, with 1 μM doxorubicin (DOX) for 16 h, with DOX only, or left untreated. Harvested cells were pelleted and processed for immunoblotting.

### Mitogenic signaling inhibition and activation

To asses mitogenic signaling activation, cells were transfected with siRNA, left to recover for 48 h and then serum-starved overnight. Next, medium with serum was added and cells were harvested at the indicated timepoints and processed for immunoblotting.

### RT-qPCR

To assess the mRNA levels after knockdown, the cells were treated with siRNA for 24 h and then cultured in standard conditions for 3 days or treated with 0.75 μM DCD and cultured 24 h after the drug washout and, subsequently, collected. Total mRNA was isolated using a Qiagen mRNeasy mini kit according to the manufacturer’s protocol. Next, reverse transcription using Anchored–oligo [30] and a Roche Transcriptor First Strand cDNA synthesis Kit (Cat no. 04 379 012 001) was performed to obtain cDNA. Quantitative PCR was performed using specific primers and a SsoAdvanced Universal SYBR Green Supermix (Bio-Rad, USA). Melting curve analysis was performed to confirm the specificity of the amplified product. Each sample was spiked with a TATAA Universal RNA Spike II control (TATAA Biocenter AB, Sweden). mRNA expression of each sample was normalized to that of the housekeeping gene *RPL30*. RT-qPCR for each sample was performed in three biological replicates.

### Fixed-cell imaging

Cells were seeded and treated when required in a glass-bottom 96-black well plate. The cells were fixed using ice cold methanol, permeabilized with 3% Triton X 100 in PBS and blocked in blocking solution (5% FBS + 0.5% Triton X 100 + 1% Na_3_N in PBS). Subsequently, the cells were incubated with primary antibody overnight at 4 °C, washed 3 × with PBS and incubated with secondary antibody and DAPI or Vybrant DyeCycle™ Green for 1 h at RT. Before imaging, the cells were washed 4 × with PBS. Imaging of fixed cells was carried out on a spinning disc system comprising an inverted Zeiss Observer.Z1 microscope, a Plan Apochromat 63 × magnification oil objective, a 40 × magnification air objective or a 20 × magnification air objective, an epifluorescence X-Cite 120 Series lamp and 473, 561 and 660 nm lasers (LaserStack, Intelligent Imaging Innovations, Inc., Göttingen, Germany), a spinning disc head (Yokogawa, Herrsching, Germany), CoolSNAP-HQ2 and CoolSNAP-EZ CCD cameras (Photometrics, Intelligent Imaging Innovations, Inc., Göttingen, Germany). SlideBook software (Intelligent Imaging Innovations, Inc., Göttingen, Germany) was used for image acquisition and analysis.

### Live-cell imaging

Cells expressing H2B-GFP were seeded in a 96-well plate at 30 000 cells per well in standard cell culture medium. Subsequently, the cells were treated with 0.75 μM DCD to induce tetraploidization. After treatment, the cells were washed with prewarmed PBS, after which FluoroBrite medium was added to proceed with live-cell imaging. Imaging was performed using an inverted Zeiss Observer Z1 microscope (Visitron Systems) equipped with a humidified chamber (EMBLEM) at 37 °C, 40% humidity and 5% CO_2_ using a CoolSNAP HQ2 camera (Photometrics) with an X-Cite 120 Series lamp (EXFO) and a Plan Neofluar 20x, or 10 × magnification air objective NA 1.0 (Zeiss, Jena, Germany). Cells were imaged for 48 h with 8-min time-lapses. Images were analyzed using Slidebook (Intelligent Imaging Innovations, Inc., Goettingen, Germany) and ImageJ (National Institutes of Health) software.

### Cell lysis and protein concentration measurement

Pelleted cells were lysed in RIPA buffer with a protease inhibitor cocktail (Pefabloc SC, Roth, Karlsruhe, Germany) and sonicated by ultrasound in a water bath for 15 min. The cell lysates were spun down at 13,600 rpm for 10 min at 4 °C using a table-top microcentrifuge (Eppendorf, Hamburg, Germany). 1 μl lysate was used to determine the protein concentration using Bradford dye at 595 nm wavelength. Subsequently, the lysates were mixed with 4 × Laemmli buffer with 2.5% ß-mercaptoethanol and boiled at 95 °C for 5 min. For fractionation, the cells were lysed in 0.1% ice-cold NP-40 and then spun down. The supernatants were transferred to separate tubes as cytoplasmic fractions and the pellets were processed using RIPA buffer to obtain nucleoplasmic fractions. The lysates were further processed as described.

### SDS-PAGE and immunoblotting

Prepped cell lysates were separated by SDS-PAGE using 10% or 12.5% gels. Protein sizes were estimated using the PrecisionPlus All Blue protein marker (Bio-Rad, USA). Gels were incubated in Bjerrum Schafer-Nielsen transfer buffer and proteins were transferred to water-activated nitrocellulose membranes (Amersham Protran Premium 0.45 NC, GE Healthcare Life Sciences, Sunnyvale, USA) using semi-dry transfer (Trans-Blot® Turbo™, Bio- Rad, USA). Next, the membranes were stained in Ponceau solution for 5 min and scanned to be used as a loading control. The membranes were blocked in 5%-10% skim milk in TBS-T (Fluka, Taufkirchen, Germany) for 1 h at RT. After blocking, the membranes were incubated with the respective primary antibodies diluted in 1% Bovine Serum Albumin (BSA) or 5% skim milk overnight at 4 °C with gentle agitation. Next, the membranes were rinsed 3 × 5 min with TBS-T, incubated 1 h at RT with HRP-conjugated secondary antibodies (R&D Systems) followed by rinsing 3 × 5 min with TBS-T. Chemiluminescence was detected using an ECLplus kit (GE Healthcare, Amersham™) and an Azure c500 system (Azure Biosystems, Dublin, USA). Protein band quantification was carried out using ImageJ software (NIH, http://rsb.info.nih.gov/ij/). All antibodies used are listed in Supplementary Table 5.

### Chromatin immunoprecipitation (CHIP)

Cells were treated with siRNA and 0.75 μM DCD (18 h treatment and release for 24 h or 48 h) after which 1 μM DOX (16 h) was added one day later. Treatments were arranged in a way that all samples were collected simultaneously. The experiments were performed using a SimpleChIP® Enzymatic Chromatin IP (Magnetic Beads) kit (Cell Signaling, #9003) according to manufacturer’s protocol. Briefly, the cells were treated in a cell culture dish with 37% PFA to fix and crosslink the proteins to the DNA. Next, the cells were collected, incubated with micrococcal nuclease and sonicated. The fractionated chromatin was subsequently incubated with an antibody (anti-p53, anti-H3, normal rabbit IgG) overnight at 4 °C with rotation. Next, magnetic beads were added to each immunoprecipitation sample and incubated for 2 h, followed by washing steps and elution. DNA was purified and used for RT-qPCR.

### Co-immunoprecipitation

Untreated and DCD-treated cells were collected and lysed in lysis buffer (25 mM Tris–HCl, pH 7.4, 150 mM NaCl, 1 mM EDTA, 1% NP-40, 5% glycerol). Magnetic beads (SureBeadsTM Magnetic Beads, Bio-Rad, USA) were conjugated with antibody (anti-USP28, anti-SPINT2, anti-Flag) according to the manufacturer ‘s protocol. Next, cell lysates were incubated with the beads overnight at 4 °C, washed 2 times with washing buffer I (25 mM Tris–HCl, pH 7.8, 500 mM NaCl, 0,5% Triton-X 100) and 3 times with washing buffer II (10 mM Tris–HCl, pH 7.8, 150 mM NaCl) and eluted with 2 × Laemmli buffer. Samples were loaded onto SDS page gels and either processed further to confirm pull-downs by immunoblotting or to detect interactors by mass spectrometry.

### FACS-based proliferation assay

Harvested cells were spun down and fixed using Fix-Perm buffer. Next, the samples were incubated with Click-it reaction mix for 30 min in the dark followed by 30 min incubation with anti-cyclin B antibody. Then, the samples were incubated for 30 min with secondary antibody. Between each incubation, the cells were washed 3 times with Perm-Wash buffer and spun down. After the incubation with secondary antibody, the cells were resuspended in PBS RNase (RNase Zap, Invitrogen, Carlsbad, USA) solution with 4',6-Diamidino-2-Phenylindole, Dihydrochloride (DAPI) and measured using an Attune Nxt acoustic focusing cytometer (Life Technologies, Carlsbad, USA).

### Mass spectrometry-based identification of interacting proteins

Mass spectrometry-based identification of USP28 was performed by Nagarunja Nagaraj at the Mass Spectrometry Core Facility of MPI Biochemistry, Martinsried, Germany, as previously described [31]. The identified proteins generated by MaxQuant V were uploaded to Perseus V 1.6.2.3. Site only, reverse and contaminant peptides were removed from the dataset and missing values were imputed using a normal distribution. Invalid values were excluded. The volcano plot function was used to identify proteins that were significantly altered using a t-test with a false discovery rate (FDR) of 0.05 and a S_0_ of 0.1.

### SPINT2 and CDKN1A mRNA expression correlation

Log-transformed gene expression values of SPINT2 and the p21 gene CDKN1A were quantified using RNA-Seq by Expectation Maximization (RSEM) (Li, 2011) with data from The Cancer Genome Atlas (TCGA) (http://cancergenome.nih.gov/). The samples comprised 285 primary colon adenocarcinoma and 41 normal tissue samples taken from colon cancer patients. Pearson’s correlation coefficient rho was used for gene expression correlation testing.

## Results

### High throughput genetic screen to identify factors involved in the arrest of tetraploid cells

Proliferation of tetraploid cells is inhibited either immediately after cytokinesis failure or after the first tetraploid mitosis (Fig. 1A). To determine factors that control the proliferation of transformed tetraploid cells, we used HCT116, a pseudo-diploid (45,X) transformed human p53-positive colorectal cancer cell line. The cells were additionally modified with Fluorescent Ubiquitination-based Cell Cycle Indicator (FUCCI, [26]). The FUCCI G_1_ sensor consists of the N-terminus of Cdt1 fused to mKO2 (Kyoto Orange fluorescent protein) that is expressed in G1 phase and degraded in S, G_2_ and M phases by the SCF complex, and the FUCCI G_2_ sensor based on N-terminus of Geminin fused to mAG (Azami Green fluorescent protein) that is degraded by the APC/C complex at the end of mitosis. To generate tetraploid cells, HCT116 cells were treated 18 h with dihydrocytochalasin D (DCD), an inhibitor of actin polymerization that prevents cytokinesis. This treatment results in a mixed population of approximately 60–70% binucleated tetraploid and 30–40% diploid HCT116 cells [17]. Under these conditions, more than 80% of both binucleated tetraploids and diploids entered the next S phase and progressed to mitosis. Whereas diploid cells proliferate normally, the subsequent mitoses of tetraploid cells were often erroneous, and about 50% of the cells arrest shortly after the second tetraploid mitosis [17]. We performed a genome-scale RNAi screen in HCT116 cells after the DCD treatment. In our settings, the diploid and tetraploid populations can be distinguished by DNA content in combination with the FUCCI sensor by image analysis of the mixed population (Fig. 1B). This strategy enabled comparing the effects of knockdown in diploids and tetraploids side-by-side in the same experiment.

To identify factors that control proliferation of tetraploids, we used an esiRNA library targeting 16,231 genes in the human genome ([28, 32, 33], Supplementary Information). Through the primary screen, we identified 432 genes whose depletion improved the proliferation of tetraploid cells; we name this category a TP53-like class, since knockdown of these genes improves the proliferation of tetraploid cells similarly to knockdown of TP53. Next, we performed a validation screen of primary TP53-like hits and confirmed 157 genes from the primary screen (Supplementary Table 1). We then calculated the Z-scores and selected genes with a Z-score > 10, resulting in a high confidence group of 90 genes (Fig. 1C). Among the strongest hits was CDKN1A (gene encoding the p21 protein), a downstream target of p53 that promotes cell cycle arrest. CDKN1A has previously been found to inhibit tetraploid proliferation, thus validating the overall strategy [16]. Pathway enrichment analysis of the hits by DAVID [34] revealed statistically significant enrichment of the pathways related to DNA replication (e.g. “DNA polymerase:primase complex”, “DNA replication initiation”), “G1-S transition of mitotic cell cycle”, “PI3k-Akt signaling pathway”, “Pathways in cancer” and “Extracellular matrix organization” (Fig. 1D, Supplementary Table 2). Comparing the list of genes identified in our screen with previous results revealed only a minor overlap with genes that were found to limit proliferation of tetraploid RPE1 cells [15]. In fact, we found a larger overlap with factors required for survival after DNA damage (Fig. 1E, Supplementary Table 3). This suggests that the factors affecting the proliferation of tetraploid cells depend on the cell type. For subsequent analyses, we selected six candidate genes that represent each category and were previously linked to colorectal cancer.

### SPINT2 and USP28 regulate proliferation of tetraploid cells

**Fig. 2:**
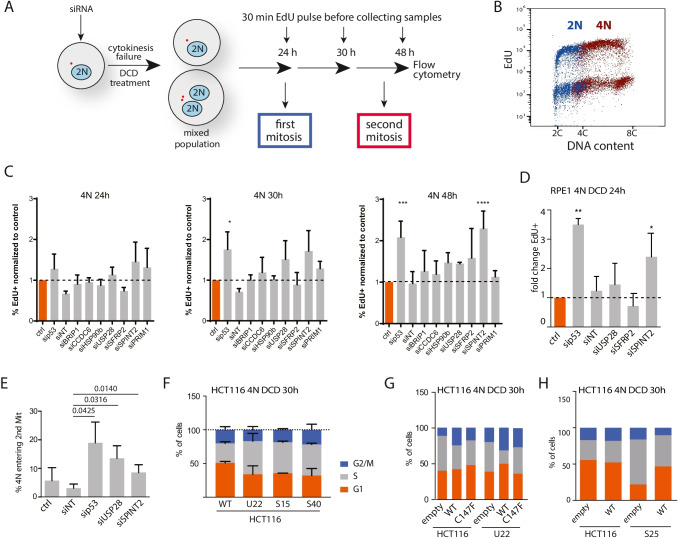
Validation of the identified targets confirms the role of USP28 and SPINT2 in the proliferation of tetraploid cancer cells. **A** Schematics of the experimental set up to validate the targets. **B** Cell cycle profile obtained by flow cytometry of EdU labeled cells after cytokinesis failure. EdU labeling allows the identification of proliferating cells; PI staining allows to distinguish cells according to their DNA content. Cell categories: diploid in G1: 2C_G1, diploid in S, G2, M: 2C_S, tetraploid in G1: 4C_G1, tetraploid in S: 4C_S and tetraploid in G2 and M: 8C_G2. C. Fold change of proliferating HCT116 cells 24 h, 30 h and 48 h after cytokinesis failure treated with siRNA against the respective genes or with a non-targeting control (siNT) compared to an untreated control (ctrl). siRNA against p53 was used as a positive control. **D** Fold change of proliferating RPE1 cells 24 h after cytokinesis failure treated with siRNA against the respective genes or with a non-targeting control compared to an untreated control. siRNA against p53 was used as a positive control. **E** Percentage of cells entering the 2^nd^ tetraploid mitosis after cytokinesis failure in HCT116 cells treated with siRNA against the respective genes or with a non-targeting control. siRNA against p53 was used as a positive control. Time-lapse live cell imaging, 8 min time frame, 72 h, 20 × air objective. At least three independent experiments, each with at least 40 cells per condition, were analyzed. **F** Cell cycle distribution analysis in tetraploids generated from USP28 and SPINT2 knockout clones (U22 and S15, S40, respectively) using flow cytometry. Knockouts were generated using CRISPR/Cas9 in HCT116 cells. Samples were analyzed 30 h after release from DCD treatment. **G** Cell cycle distribution analysis in tetraploids generated from USP28 knockout clone U22 after transfection with a control empty plasmid, a plasmid with wildtype USP28 and a plasmid containing a mutant USP28 lacking the deubiquitinase active site (C147F). Representative plots of three biological experiments are shown. **H** Cell cycle distribution analysis in tetraploids generated from SPINT2 knockout clone S25 after transfection with a control empty plasmid or a plasmid with wildtype SPINT2. Representative plots of three biological experiments are shown

For validation and functional follow up, we selected one factor from each significantly enriched category: SFRP2 (Secreted Frizzled Related Protein 2), coding for a soluble modulator of WNT signaling, HSP90AB1 (Heat Shock Protein 90 Alpha Family Class B Member 1) coding for a molecular chaperone, CCDC6 (Coiled-Coil Domain Containing 6), an uncharacterized putative tumor suppressor, SPINT2 (Serine Peptidase Inhibitor, Kunitz Type) coding for a hepatocyte growth factor inhibitor, BRIP1 (BRCA1 Interacting Protein C-Terminal Helicase 1), a breast cancer-associated gene coding for a protein involved in DNA repair, PRIM1, a catalytic subunit of the DNA primase complex and component of the DNA polymerase alpha complex, and USP28 (Ubiquitin Specific Peptidase 28), a deubiquitinase involved in DNA damage response checkpoint and MYC proto-oncogene stability (Fig. 1D). To validate the selected candidates, we treated the cells with siRNAs for the respective genes, followed by DCD to induce cytokinesis failure. Samples collected at defined time points were treated for 30 min with EdU (5-ethynyl-2’-deoxyuridine), which incorporates into the replicating DNA, to determine the fraction of proliferating cells (Figs. 2A, S1A). Subsequent flow cytometry was used to quantify the proportion of proliferating diploids and tetraploids and their cell cycle phases (Figs. 2B, S1A). We found that treatment with the respective siRNAs depleted on average 80% of the candidate proteins (Fig. S1B). Knockdown of SPINT2 and USP28 improved tetraploid proliferation, thus confirming their role in the cellular response to tetraploidy. Depletion of the other factors did not affect the proliferation of tetraploids and they were excluded from further analysis (Fig. 2C). No effect was observed in diploids (Fig. S1C). Analysis in RPE1 cells confirmed the role of p53 and SPINT2 in tetraploid proliferation as has been shown previously, but we observed no effect of USP28 on the survival of tetraploid RPE1 cells (Fig. 2D). Time-lapse live cell imaging further validated that depletion of SPINT2 and USP28 increased the number of tetraploid HCT116 cells that entered a second mitosis after cytokinesis failure (Fig. 2E). Finally, we used CRISPR/Cas9 to knock out SPINT2 and USP28 in HCT116 cells (Fig. S1D, E). The knockout cell lines showed an improved proliferation upon induced cytokinesis failure, which was abolished upon transfection with plasmids carrying the respective wild type genes (Figs. 2F-H, S1F, G). Finally, we co-depleted the analyzed factors together with TP53. We observed no additive effect with TP53 depletion, as the fraction of proliferating tetraploid cells was similar in double depletions as in single depletions (Fig. S2). This suggest that both USP28 and SPINT2 act in the same signaling pathways as p53.

**Fig. 3:**
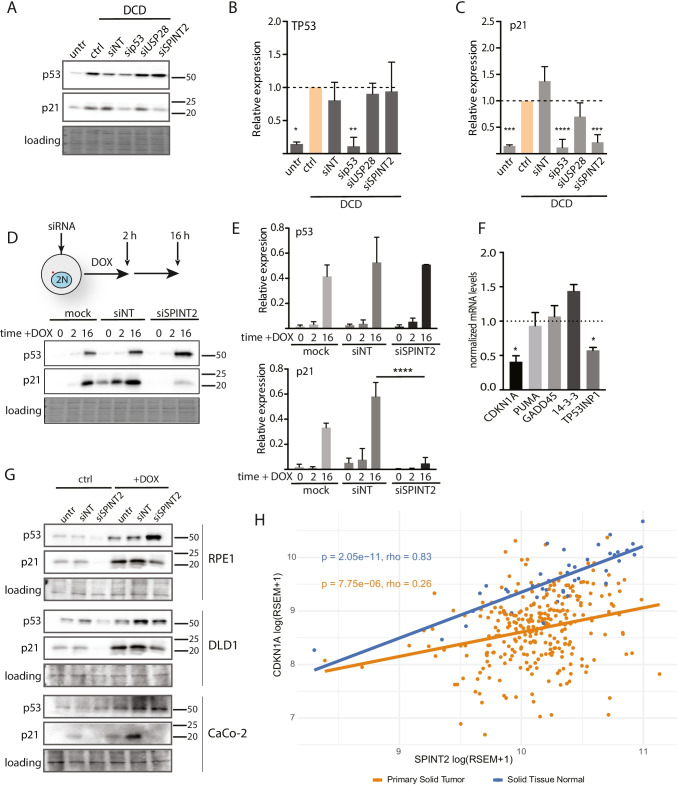
SPINT2 modulates the expression of p21 independent of p53 level. **A** Expression levels of p53 and p21 upon depletion of the candidate factors in DCD treated HCT116 cells. Ponceau staining was used as a loading control. **B**, **C** Quantification of p53 and p21 levels upon depletion of the candidate factors in DCD treated cells. Mean and SEM of three experiments are shown. T test was used for statistical evaluation. **D** Schematic presentation of the experiment and expression of p53 and p21 in doxorubicin (DOX) treated cells depleted for SPINT2. Ponceau staining was used as a loading control. **E** Quantification of p53 and p21 levels upon SPINT2 depletion in DOX treated cells. Mean and SEM are shown. T test was used for statistical evaluation. **F** Normalized mRNA levels of p53 downstream targets upon SPINT2 depletion in DOX treated cells. Mean and SEM of at least three independent experiments are shown, normalized to control RPL30. **G** Immunoblots of p53 and p21 in RPE1, DLD1 and CaCO2 cells upon SPINT2 depletion. Ponceau staining was used as a loading control. **H** Correlation of CDKN1A and SPINT2 mRNA expression levels in colorectal adenocarcinoma and in normal tissues. RSEM (RNA-Seq by Expectation Maximization) quantification of RNA abundance derived from RNA-seq data

Formation of tetraploid cells activates the p53 response and induces the expression of p21 in most human cell lines [13]. We therefore tested whether the depletion of SPINT2 and USP28 diminishes p53 activity, thereby facilitating the growth of tetraploid cells. While the p53 levels were not affected by SPINT2 and USP28 depletion, the expression of p21 was strongly reduced upon SPINT2 depletion and partly also upon USP28 depletion (Fig. 3A-C). Activation of Hippo signaling and PIDDosome has previously been shown to control the survival of tetraploid cells [15, 22]. Since our screen did not identify any members of these two pathways, we asked whether they were activated in response to tetraploidy in HCT116 cells. Indeed, we found that MDM2 and caspase 2 became cleaved in response to cytokinesis failure in HCT116 cells, demonstrating that PIDDosome activity increased upon whole genome doubling as previously described (Fig. S3A, B). Importantly, depletion of SPINT2 or USP28 did not affect MDM2 or caspase 2 cleavage (Fig. S3B). In contrast, YAP localization was not altered and the phosphorylation of LATS2 was not increased in tetraploid HCT116 cells, suggesting that cytokinesis failure in these cells does not activate the Hippo pathway (Fig. S3C-E). We conclude that SPINT2 and USP28 affect the proliferation of tetraploid HCT116 cells independent of Hippo or PIDDosome signaling.

### SPINT2 regulates the expression of a subset of TP53 targets

SPINT2, which is a putative tumor suppressor, encodes a transmembrane protein with two extracellular protease inhibitor domains (Kunitz domains) that act on a variety of serine proteases [35]. It inhibits the binding of signaling molecules, such as HGF (hepatocellular growth factor) to the c-Met receptor and, thereby, affects the ERK, AKT and STAT3 pathways (Fig. S4A). In diploid HCT116 cells, loss of SPINT2 also enhanced activation of the AKT pathway in response to growth factors (Fig. S4B). Upon the transfer of starving cells to serum-proficient medium, the phosphorylation of AKT was significantly increased, whereas the expression of p21 was nearly abolished in cells with reduced SPINT2 expression (Fig. S4B, C). Moreover, the nuclear levels c-Myc, a pro-proliferative transcription factor and c-MET target (via STAT3) [36], were found to be increased significantly upon SPINT2 knockdown (Fig. S4D). For future studies, it will be interesting to investigate whether loss of the c-MET inhibitor SPINT2 indeed bypasses cell cycle arrest in tetraploid cells through the activation of pro-proliferative factors.

A striking result of SPINT2 depletion was a reduction in CDKN1A (p21) expression despite p53 activation (Fig. 3A-C), which is likely the main reason for improved tetraploid proliferation. To determine whether SPINT2 depletion reduces CDKN1A expression also in response to other cellular stresses, we treated diploid cells with the topoisomerase II inhibitor doxorubicin (DOX). This agent causes DNA damage that activates the p53-mediated response and results in p53 stabilization and increased p21 levels. We found that SPINT2 depletion abolished CDKN1A activation upon DOX treatment, while the p53 levels were not affected, similar to the response to tetraploidy (Fig. 3D-F). Using RT‐qPCR, we evaluated the effect of SPINT2 depletion on the expression of p53 targets upon treatment with DOX. We found that the expression of factors required for apoptosis, such as GADD45 or PUMA, were not affected by SPINT2 depletion. In contrast, we found that the expression of factors that promote cell cycle arrest, i.e., CDKN1A and TP53INP1, was reduced in response to DNA damage when SPINT2 was depleted (Fig. 3F). The reduced CDKN1A expression upon SPINT2 depletion was also observed in RPE1, DLD1 and CaCo2 cells (Fig. 3G). Finally, gene expression data from The Cancer Genome Atlas database (TCGA) revealed a strong correlation between the CDKN1A and SPINT2 expression levels in healthy samples, and a weak correlation in cancer samples in which SPINT2 is frequently mutated (Fig. 3H) [37]. This suggests that SPINT2 regulates CDKN1A expression.

**Fig. 4:**
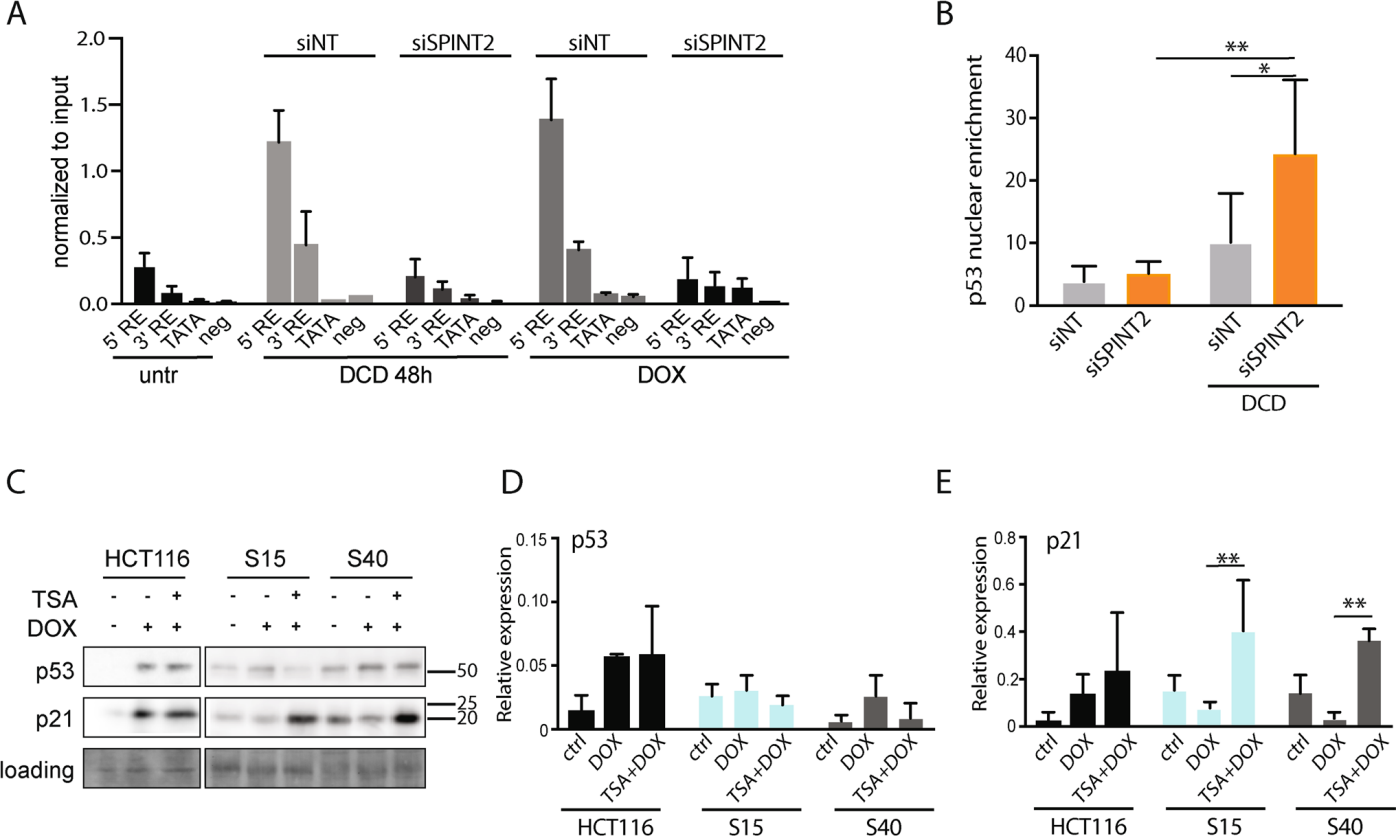
Effect of SPINT2 on CDKN1A (p21) expression is mediated via promotor acetylation. **A** Chromatin immunoprecipitation of p53 with defined regulatory elements within the *CDKN1A* promoter. **B** Nuclear enrichment of p53 after siRNA knockdown of SPINT2. NT: non-targeting siRNA control. Mean and SD of three independent experiments are shown. T test, * *p* < 0.05; ** *p* < 0.01. **C** Representative immunoblot of p53 and p21 in HCT116 and two SPINT2 knockout clones, S15 and S40, after TSA and doxorubicin (DOX) treatment. Ponceau staining was used as a loading control. **D**, **E** Quantification of p53 and p21 levels from three independent experiments normalized to the loading control. Mean with SD is shown. T test. * *p* < 0.05; ** *p* < 0.01

To investigate how SPINT2 affects CDKN1A expression, we performed chromatin immunoprecipitation of p53 with the defined regulatory elements of the *CDKN1A* promoter. We found that the binding of TP53 to the *CDKN1A* promoter was lost upon SPINT2 depletion in DOX or DCD treated cells (Fig. 4A). This was not due to a lack of p53 in the nucleus (Fig. 4B). Histone acetylation within the *CDKN1A* promoter region is essential for its expression [38]. We tested whether inhibition of histone deacetylases (HDACs) restores the expression of *CDKN1A* in cells lacking SPINT2 expression. To this end, we used SPINT2 knockout HCT116 cells. We treated these cells with trichostatin A (TSA), a potent HDAC inhibitor, simultaneously with DOX. We found that the loss of SPINT2 no longer suppressed the expression of p21 upon HDAC inhibition (Fig. 4C-E). To validate our finding in other cell lines, we used DLD-1 and RPE1 and knocked down SPINT2 using siRNA. After treatment with TSA and DOX, we indeed found that loss of SPINT2 no longer suppressed the expression of p21 upon HDAC inhibition (Fig. S5A,B). We conclude that SPINT2 is a previously unidentified regulator of CDKN1A expression in human cells that affects histone modifications within the *CDKN1A* promoter.

### Loss of USP28 expression affects centrosome clustering

USP28 has recently been identified as a factor that together with 53BP1 and TRIM37 stabilizes p53 in response to centrosomal stress or after extended duration of mitosis in RPE1 cells [39–41], whereas no effect of USP28 was observed after cytokinesis failure in RPE1 cells. This finding is in line with our observations (Fig. 2D). Indeed, we found that the function of USP28 in tetraploids differs from its effect after centrosomal depletion, since 53BP1 did not influence the proliferation of tetraploid HCT116 cells (Fig. S6A). Moreover, although mitosis in tetraploids generally takes longer than that in diploids, it only rarely exceeds 90 min (Fig. S6B). Because most RPE1 cells arrest in the G1 phase immediately following cytokinesis failure, while HCT116 cells progress through 2–3 cell cycles before arresting, we hypothesized that USP28 may be required for the response of proliferating tetraploid HCT116 cells, which cannot be readily observed in primary cell lines.

**Fig. 5:**
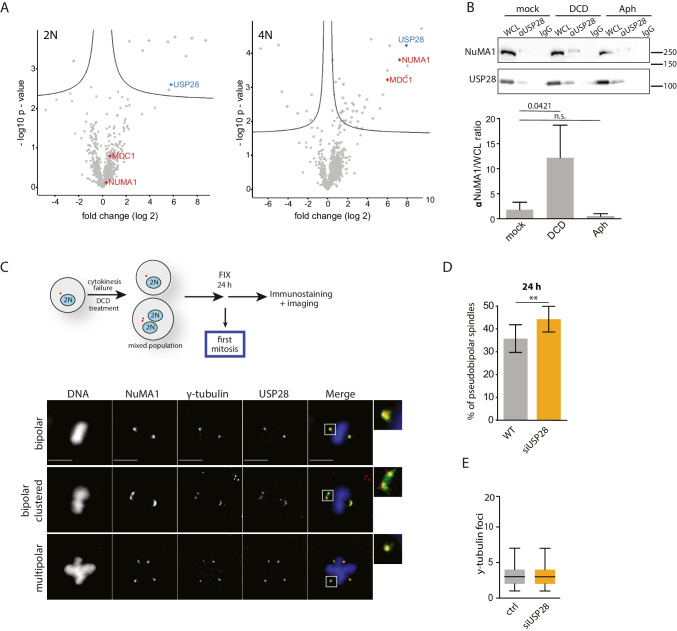
USP28 affects centrosome clustering in proliferating tetraploids. **A** Volcano plot showing the interaction partners of USP28 (blue) in diploid and tetraploid HCT116 cells. The interactors NuMA1 and MDC1 are marked in green. Complete results are provided in Supplementary Table 4. **B** Immunoblot of USP28 and NuMA1 after co-immunoprecipitation of USP28 using magnetic beads (upper panel). Ratio between NuMA1 and respective whole-cell lysates (lower panel). Samples were treated with DCD and aphidicolin (Aph), respectively. **C** Schematics of the experiment and immunofluorescence pictures in HCT116 γ-tubulin-mRuby cells stained for USP28 and NuMA1 showing localization of USP28 and NuMA1 in relation to γ-tubulin. DNA was stained using DAPI. Scale bars: 10 µm. **D** Percentage of pseudo-bipolar spindles in tetraploid HCT116 γ-tubulin-mRuby cells and after USP28 siRNA knockdown. Cells were fixed 24 h after DCD washout and analyzed by immunofluorescence. T test was used for statistical evaluation. **E** Analysis of centrosome numbers in tetraploid HCT116 γ-tubulin-mRuby cells and after USP28 siRNA knockdown. T test was used for statistical evaluation

To elucidate the function of USP28 in response to tetraploidy, we performed immunoprecipitation (IP) of proteins interacting with USP28 followed by mass spectrometry. Specifically, we analyzed HCT116 cells with and without DCD treatment, looking for interactors 24 h after cytokinesis failure. We found several proteins whose presence upon co-IP with USP28 was significantly increased in the DCD-treated samples (Fig. 5A). Among them was the mediator of DNA damage checkpoint 1 (MDC1), a previously identified USP28 interactor upon DNA damage required for full activation of the DNA damage checkpoint [27]. Interestingly, we also found interaction with nuclear mitotic apparatus protein 1 (NuMA1) which is involved in spindle apparatus and microtubule functions and has not previously been related to USP28. The co-IP of USP28 and NuMA1 was specific for tetraploid cells, as confirmed by a pull down followed by immunoblotting (Fig. 5B). Both USP28 and NuMA1 also colocalized on centrosomes (Figs. 5C, S6C).

One of the well-recognized functions of NuMA1 is its involvement in clustering of supernumerary centrosomes [42]. We asked whether the USP28-NuMA1 interaction might regulate centrosome clustering in tetraploid cells. Immunofluorescence microscopy revealed that HCT116 tetraploid cells clustered the spindle poles more efficiently in the absence of USP28 than in control cells (on average 45%, compared to 35%, Fig. 5D), while the numbers of centrosomes were not altered (Fig. 5E). Since pseudo-bipolar mitosis leads to less cellular death than multipolar mitosis [21], this increased centrosomal clustering may improve the viability of tetraploid cells. USP28 is a de-ubiquitinase and regulates the stability of several proteins. However, we did not observe any difference in NuMA1 abundance upon USP28 depletion and, thus, the mechanism underlying the effect of USP28 on centrosome clustering remains unclear (Fig S6D, E).

### Increased DNA damage in tetraploid cells

**Fig. 6:**
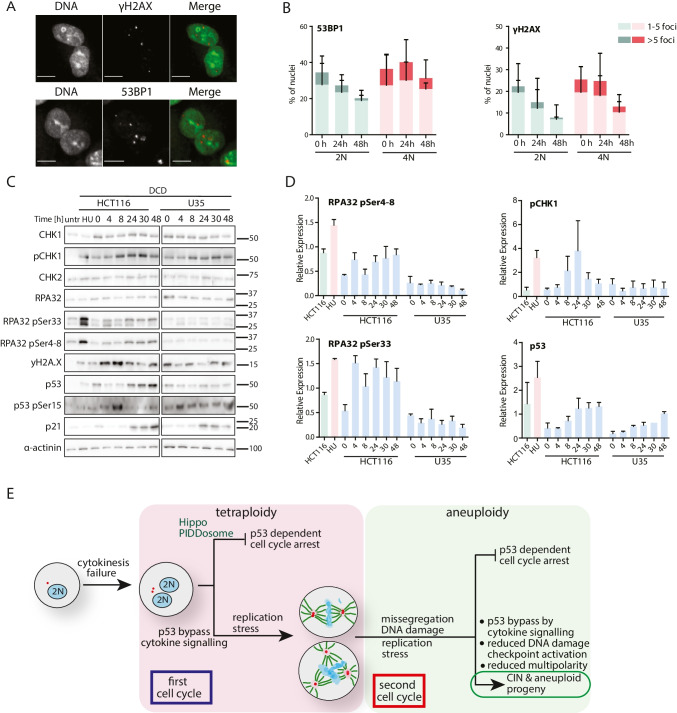
DNA damage signaling in tetraploid cells is reduced upon loss of USP28 expression. **A** Representative immunofluorescence images of 53BP1 (left) and γH2AX (right) foci in binucleated tetraploid HCT116 cells. **B** Quantification of 53BP1 (left) and γH2AX (right) foci in diploid (green) and tetraploid (red) HCT116 cells. Mean ± SEM is shown. **C** Immunoblot showing the time course of DNA damage checkpoint proteins after DCD treatment in HCT116 cells (left) and after USP28 knockout (right). α-actinin was used as a loading control. **D** Relative expression of DNA damage-associated proteins in DCD treated HCT116 cells with and without USP28. Values were normalized to the loading control. Mean ± SEM is shown. **E** Schematic depiction of the cellular fate after cytokinesis failure

Another interactor of USP28 specifically enriched in tetraploid cells was MDC1, a facilitator of DNA damage response and checkpoint activation [27]. Previously, it has been suggested that WGD triggers DNA damage in proliferating cells, as has been shown in *Drosophila* and U2OS cells [23, 43]. Imaging of DNA damage markers 53BP1 and yH2AX in binucleated tetraploid HCT116 cells showed an increased accumulation of DNA damage and its delayed repair compared to that in diploids (Fig. 6A, B). Immunoblotting revealed an increased phosphorylation of RPA32 at Ser33 and, with a delay, at Ser4/8 (targets of ATR and DNA-PK, respectively [44], Fig. 6C,D). The yH2AX signal that marks DNA damage also increased during the 48 h time course after cytokinesis failure, as well as the phosphorylation of CHK1 and the accumulation of p53 and p21 (Figs. 6B, C, S7). Depletion or knockout of USP28 diminished the checkpoint activation and accumulation of p53 and p21 (Figs. 6C, D, S7). The time course data showed that the DNA damage accumulates when tetraploid cells enter the cell cycle and further proliferate. We conclude that tetraploidy leads to increased DNA damage and that loss of USP28 alleviates subsequent checkpoint activation, thereby enhancing the proliferation of tetraploid cells.

## Discussion

Whole genome doubling shapes the evolution of cancer and drives transformation, metastasis and drug resistance, yet human non-transformed cells usually arrest after becoming tetraploid, which raises the question how proliferating tetraploid populations arise. We performed a genome-scale screen for factors enabling proliferation of a transformed cell line, in which we induced cytokinesis failure. These cells do not arrest immediately after becoming tetraploid, but rather progress through the next cell cycles and only later become trapped in an irreversible arrest, likely due to accumulation of genomic abnormalities. We show that three factors are essential for survival of tetraploid cells in this context: increased mitogenic signaling and reduced expression of cell cycle inhibitors, the ability to establish bipolar spindles, and reduced DNA damage signaling (Fig. 6E).

Using an esiRNA library, we identified 140 genes whose depletion improved the proliferation of tetraploid cells, of which only two factors have been previously identified in other screens. Validation of selected candidates confirmed that USP28 and SPINT2 negatively regulate the proliferation of tetraploids, acting independently of the Hippo and PIDDosome pathways that were previously identified to inhibit tetraploid proliferation [15, 22]. Our results show that the cellular response to tetraploidy is cell type and context dependent and that a complete picture of the molecular processes affected by tetraploidy is still missing.

SPINT2, a putative tumor suppressor, was identified as a factor limiting the proliferation of tetraploids not only in HCT116 cells, but also in RPE1 cells [15]. SPINT2 encodes an inhibitor of growth factor signaling and, thus, the increased proliferation rates upon SPINT2 loss may be explained by overactivation of growth factor signaling, which in turn leads to a bypass of the p53-dependent cell cycle arrest [15, 35]. In tetraploid cells, both activation of mitogenic signaling by loss of SPINT2 expression and overexpression of c-Myc and cyclin D1 or D2 (downstream targets of the mitogenic signaling) lead to increased proliferation [15, 24, 25]. Our data suggest that SPINT2 modulates CDKN1A transcription independently of p53. Depletion of SPINT2 reduced CDKN1A transcription upon induced cytokinesis failure as well as upon induction of DNA damage, possibly via epigenetic regulation. In agreement with this observation, we found that the expression levels of SPINT2 and CDKN1A strongly correlated in healthy human cells. Interestingly, the correlation was found to be reduced in tumor samples. This may reflect alterations in the regulation of these factors, which occur during cellular transformation. Since SPINT2 has been reported to act as a putative tumor suppressor in some cancer types, our findings suggest the mechanism of its tumor suppressing activity.

We found that loss of USP28, a deubiquitinase, also increased the proliferation of tetraploid HCT116 cells. USP28 was recently identified as a key factor affecting proliferation upon centrosome loss in RPE1 cells, but not after cytokinesis failure [39–41]. USP28 also interacts with 53BP1 to arrest cells upon centrosome loss and prolonged mitosis [39–41], but 53BP1 plays no role in the proliferation of tetraploid HCT116 cells. We propose that the observed difference between RPE1 and HCT116 is due to a different timing of the cell cycle arrest after tetraploidization. While primary cells mostly arrest in the first G1 phase immediately after cytokinesis failure, cancer cells progress through at least one cell cycle with doubled chromosome and centrosome numbers [17]. Thus, USP28 loss increases the proliferation of tetraploid cells by diminishing the negative impact of tetraploidy in subsequent cell cycles.

To investigate how USP28 affects the proliferation of tetraploids, we identified proteins interacting with USP28 specifically after cytokinesis failure, and found among them, most prominently, MDC1 and NuMA1. NuMA1, a specific USP28 interactor after cytokinesis failure, plays multiple roles in ensuring mitotic spindle integrity. It is recruited to the minus-ends of microtubules and acts as an adaptor for dynein and dynactin to allow spindle pole organization by generating pulling forces that control the spindle position [45]. Although loss of USP28 did not affect the protein level of NuMA1 nor its localization, it increased the clustering of extra centrosomes and, thus, reduced multipolar mitoses. High levels of NuMA1 in tumor cells lead to high rates of multipolar mitoses and, conversely, depletion of NuMA1 leads to increased clustering of multiple centrosomes [42]. As USP28 and NuMA1 likely interact in mitotic cells and USP28 depletion did not have an impact on NuMA1 protein levels, our data suggest a new ubiquitin-dependent mechanism that may be involved in the control of clustering of extra centrosomes. Recent evidence suggests a function of NuMA1 also in the cellular response to DNA damage [46]. Future research will show whether this novel function of NuMA1 also contributes to the cellular response to tetraploidy. MDC1, a key mediator of the DNA damage response and replication checkpoint, was found to interact with USP28 specifically in tetraploid cells. USP28 is a substrate for ATM in the DNA damage response that stabilizes Chk2 and 53BP1 [27]. Previously, accumulation of the replication stress marker 53BP1 was observed in a human cancer cell line after whole genome doubling. Similarly, we observed increased phosphorylation of RPA32 (both Serine 4-8, a target of DNA-PK and ATM kinases, and Serine 33, target of ATR[44]) and CHK1, which also suggests increased replication stress and DNA damage response in tetraploid cells. Loss of USP28 diminished this signaling. Additional markers of replication stress and DNA damage signaling, such as ATM or CHK2 phosphorylation, should be evaluated in order to reveal the exact nature of the DNA damage. Scattered evidence suggesting that tetraploid cells suffer from increased DNA damage has been reported before [17, 23, 43], although the source remains unclear. On one hand, tetraploidy can trigger replication stress by a so far unidentified mechanism. On the other hand, it is possible that cells that do not arrest immediately after cytokinesis failure undergo erroneous mitoses. Consequently, daughter cells inherit imbalanced chromosome numbers and become aneuploid, which may lead to further replication stress and increased DNA damage [47]. Oxidative DNA damage also increases upon tetraploidization, which may again contribute to increased genomic instability [17]. Finally, two recent publications showed that the two nuclei in binucleated human cancer cells or in *Drosophila* after cytokinesis failure replicate asynchronously and accumulate DNA damage [23, 43]. We propose that replication stress and DNA damage response play important roles in limiting the proliferation of tetraploid cells. Further investigation of how tetraploidy increases DNA damage will be important to understand how whole genome doubling contributes to the evolution of cancer genomes.

## Supplementary Information

Below is the link to the electronic supplementary material.

Supplementary file 1 Summary results of the screen. (XLSX 26 KB)

Supplementary file 2 Pathway analysis of the identified candidates. (XLSX 32 KB)

Supplementary file 3 Comparison with other screen results. (XLSX 15 KB)

Supplementary file 4 Results of the mass spectrometry of USP28 pull down. (XLSX 23 KB)

Supplementary file 5 List of used antibodies. (DOCX 16 KB)

Supplementary file 6 (PDF 8794 KB)

## Data Availability

All data sets generated during the study are available in the Supplementary tables.
