# Supplementary material for: Loss of USP28 and SPINT2 expression promotes cancer cell survival after whole genome doubling
Source: Cell Oncol (Dordr). 2021 Dec 28;45(1):103–19. doi: 10.1007/s13402-021-00654-5 (PMC8881269; doi:10.1007/s13402-021-00654-5)
Supplement: Supplementary file 5 — List of used antibodies. (DOCX 16 KB) [file 13402_2021_654_MOESM5_ESM.docx]

**Supplementary table 5** List of used antibodies

| **Antigen** | **Cat. No.** | **Host** | **Application** | **Reference/Company** |
| --- | --- | --- | --- | --- |
| Akt | 9272 | Rabbit | WB, 1:1000 | Cell Signaling |
| p-Akt (Ser473) | 4060 | Rabbit | WB, 1:1000 | Cell Signaling |
| HAI-2 (SPINT2) | PA5-19360 | Goat | WB, 1:200 | Thermo Fischer |
| HAI-2 (SPINT2) | AF1106 | Goat | IP, 1:200 | R&D Systems |
| MDM2, clone IF2 | MABE340 | Mouse | WB, 1:400 | Merck Millipore |
| Cyclin D2 | 3741 | Rabbit | WB, 1:1000 | Cell Signaling |
| Caspase 2 | ALX-804-356-C100 | Rat | WB | Enzo |
| Histone H3 (D2B12) XP | 4620 | Rabbit | CHIP, 2ug | Cell Signaling |
| Normal Rabbit IgG | 2729 | Rabbit | CHIP, 2ug | Cell Signaling |
| c-myc | D84C12 | Rabbit | WB, 1:1000 | Cell Signaling |
| Cyclin D1 | 2978 | Rabbit | WB, 1:1000 | Cell Signaling |
| LATS | 5888 | Rabbit | WB, 1:1000 | Cell Signaling |
| p-LATS | 8654 | Rabbit | WB, 1:1000 | Cell Signaling |
| YAP | 14074 | Rabbit | WB, 1:1000 | Cell Signaling |
| p-YAP | 13008 | Rabbit | WB, 1:1000 | Cell Signaling |
| USP28 | Ab126604, Ab70893 | Rabbit | WB, 1:1000 | Abcam |
| NUMA1 | Sc-365532 | Mouse | WB, 1:500, IF 1:500 | Santa Cruz |
| GAPDH | 2118 | Rabbit | WB, 1:1000 | Cell Signaling |
| anti-rabbit HRP | HAF008 | Goat | WB, 1:5000 | R&D Systems |
| anti-mouse HRP | HAF007 | Goat | WB, 1:5000 | R&D Systems |
| anti-goat HRP | HAF019 | Goat | WB, 1:5000 | R&D Systems |
| p53 | Sc-126 | Mouse | WB, 1:500, CHIP 2ug | Santa Cruz |
| Chk1 | Ab32531-100 | Rabbit | WB, 1:1000 | Abcam |
| Phospho-Chk1 (Ser345) | S345 | Rabbit | WB, 1:1000 | Cell Signaling |
| RPA32 | Ab2175 | Mouse | WB, 1:1000 | Abcam |
| Phospho-RPA32 (S33) | A300-246A | Rabbit | WB, 1:1000 | Bethyl |
| Phospho-RPA32 (S4/S8) | A300-245A | Rabbit | WB, 1:1000 | Bethyl |
| Phospho-p53 (Ser15) | 9284T | Rabbit | WB, 1:500 | Cell Signaling |
| Phospho-p53 (Ser15) | sc-101762 | Rabbit | WB, 1:1000 | Santa Cruz |
| CDKN1N | 2947S | Rabbit | WB, 1:1000 | Cell Signaling |
| Phospho-H2A.X (Ser139) | 05-636 | Mouse | WB, 1:500 | Merck Millipore |
| Phospho-H2A.X (Ser139) | Ab2893 | Rabbit | WB, 1:1000 | Abcam |
| Cyclin B1 | 4135S | Mouse | WB, 1:1000 | Cell Signaling |
| Cyclin B1 | 05-373 | Mouse | FACS, 1:400 | Merck Millipore |
| Cyclin A1 | Sc-271682 | Mouse | IF, 1:500 | Santa Cruz |
| α-actinin | Sc-17829 | Mouse | WB, 1:1000 | Santa Cruz |
| 53bp1 | sc-22760 | Rabbit | IF, 1:500 | Santa Cruz |
