# Supplementary material for: Loss of USP28 and SPINT2 expression promotes cancer cell survival after whole genome doubling
Source: Cell Oncol (Dordr). 2021 Dec 28;45(1):103–19. doi: 10.1007/s13402-021-00654-5 (PMC8881269; doi:10.1007/s13402-021-00654-5)
Supplement: Supplementary file 6 — (PDF 8794 KB) [file 13402_2021_654_MOESM6_ESM.pdf]

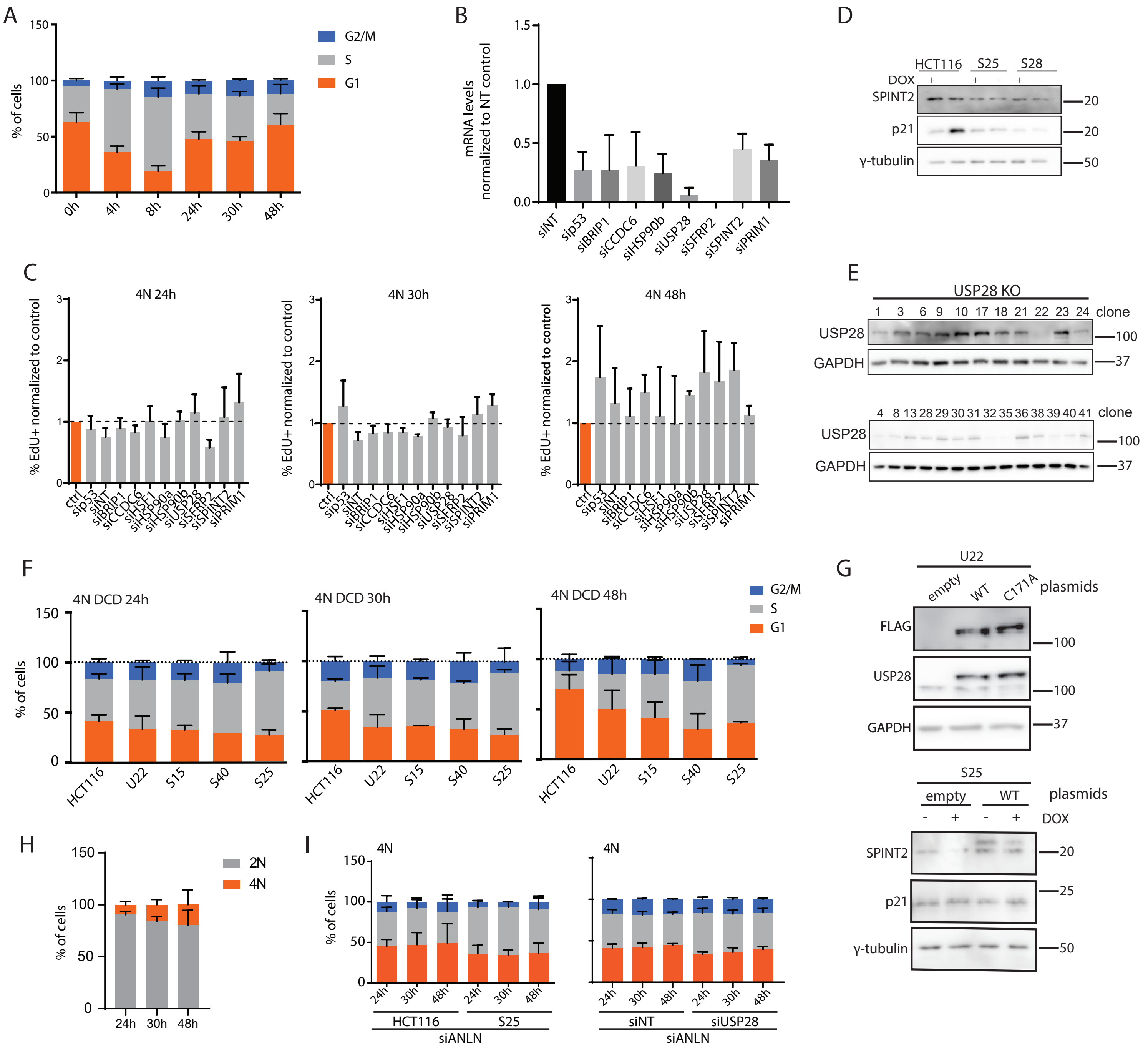

Supplementary Figure 1 Validation of selected candidates

(A) Cell cycle profile after a release from DCD in HCT116.

(B) Efficiency of siRNA depletion evaluated by q-RT-PCR. Note that the yield of q-RT-PCR of SFRP2 was near or below detection limit in depleted samples.

(C) Proliferation evaluated as fold changes of EdU incorporation in diploid cells after depletion of respective factors. siNT - non targeting control.

(D) Example of an immunoblot for validation of the SPINT2 knock out. Sequencing of the SPINT2 confirmed a short deletion within the exon 2. The knock out cell lines also lost the ability to induce p21 expression. Four clones were obtained: S15, S25, S28 and S40.

(E) Example of an immunoblot for validation of the USP28 knock out. Two clones were obtained: U22 and U35.

(F) Examples of cell cycle profiles in the knock out cell lines 24, 30 and 48 h after induced cytokinesis failure.

(G) Validation of the overexpression of USP28 and SPINT2, respectively, in knock out cell lines. Empty - an empty control vector, WT: a vector carrying the wt gene; C171A - vector carrying the mutant version lacking the deubiquitinase activity. DOX - doxorubicin.

(H) Cell cycle profiles after anilin depletion in HCT116 and S25 (left) and in USP28 depleted HCT116 (right).

(I) Percentage of diploid (2N) and tetraploid (4N) populations after anilin knock down at indicated timepoints.

A

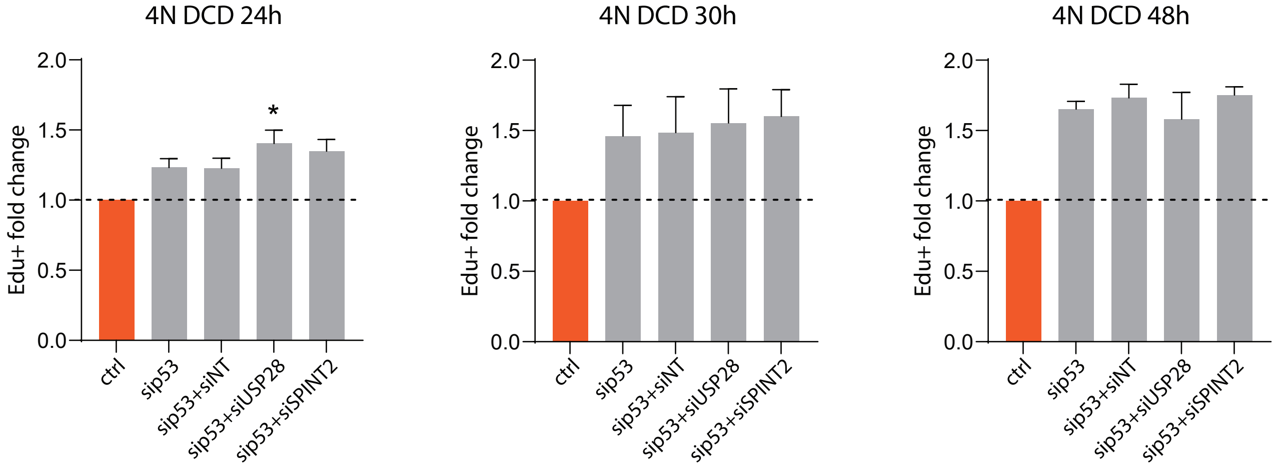

Supplementary Figure 2 Double knockdown of the candidate genes with TP53

Proliferation evaluated as fold changes of EdU incorporation in tetraploid cells after depletion of p53 alone or together with the other factors analyzed factors. Proliferation of all knock downs is significantly increased when compared to the control sample. No significant difference was detected between single and double knock downs, with exception of double knockdown of *TP53* and *USP28* 24 h after the release from DCD treatment. siNT - non targeting control

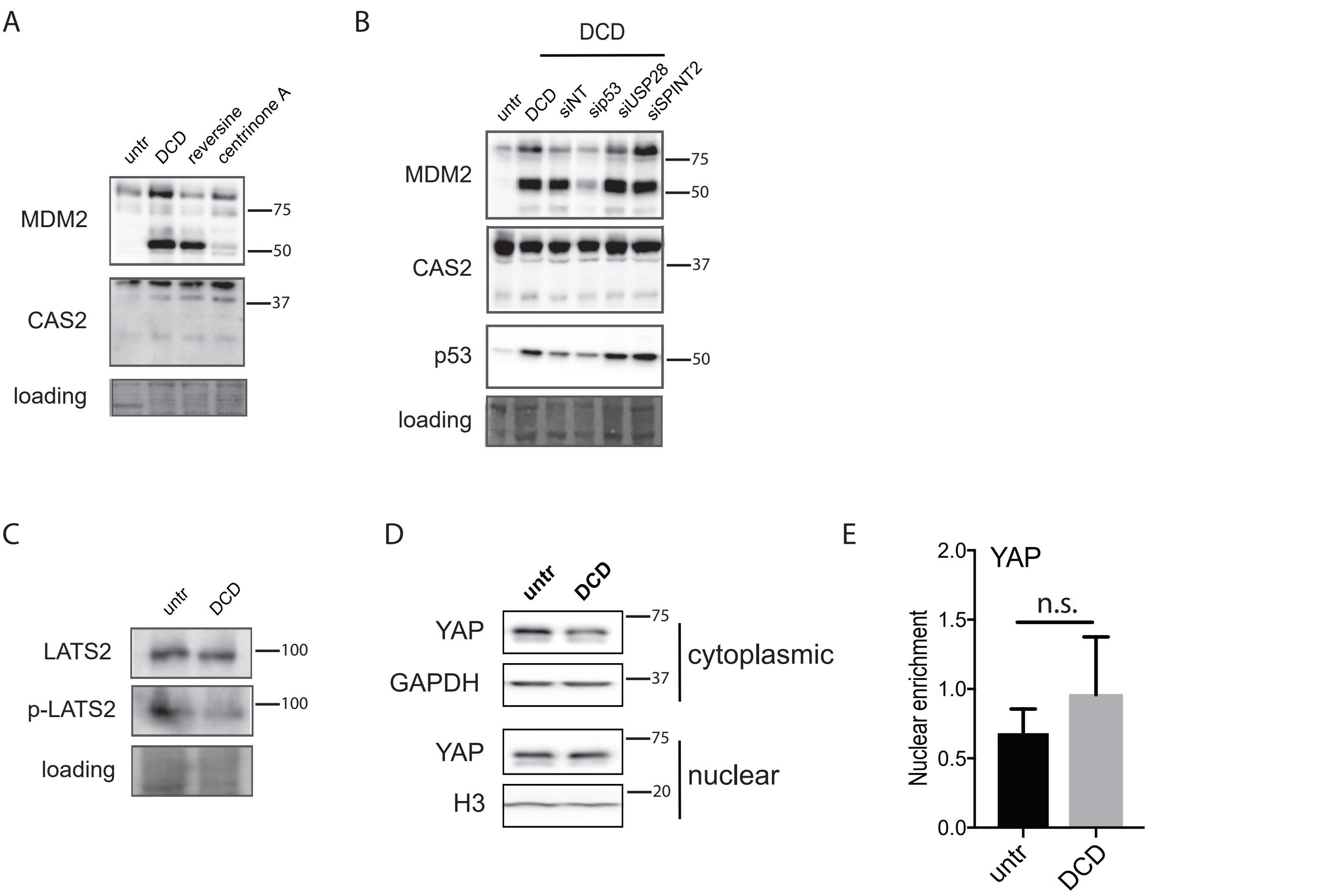

Supplementary Figure 3 Activation of PIDDosome and Hippo pathway in tetraploid HCT116 cells

(A) MDM2 and CAS2 cleavage in HCT116 upon treatments with DCD, centrinone and reversine, respectively.
(B) MDM2 and CAS2 cleavage in HCT116 upon depletion of p53, USP28 and SPINT2.
(C) Phosphorylation of LATS2 in HCT116 cells untreated or treated with DCD for 18 h.
(D, E) Immunoblot and quantification of YAP nuclear enrichment in HCT116 upon DCD treatment. Mean and SEM of three independent experiments is shown.

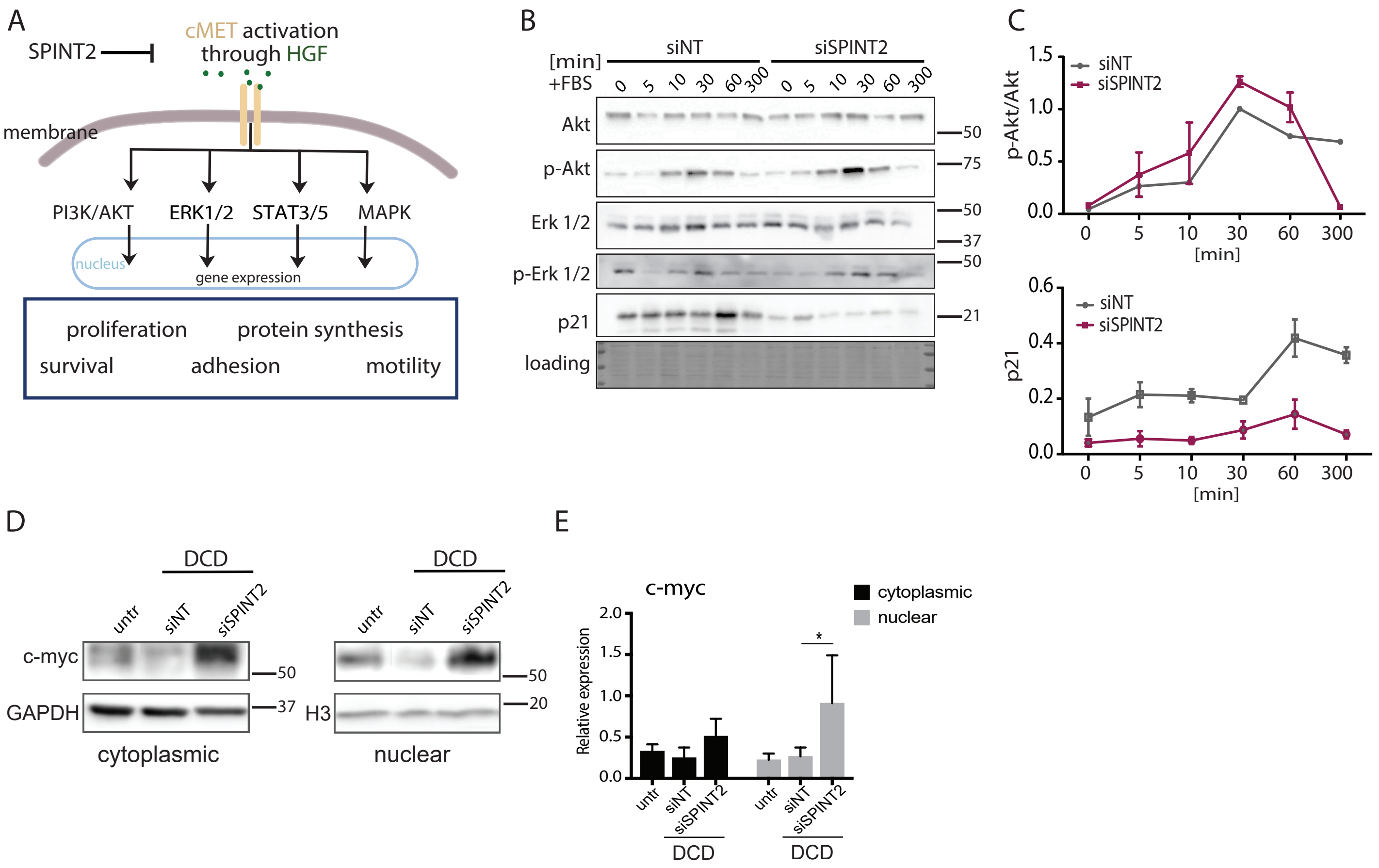

Supplementary Figure 4 Activation of the cMET pathway in tetraploid HCT116 cells

(A) Schematic depiction of the cMET pathway.
(B) Representative immunoblot of cMET downstream kinases upon a release from starvation.
(C) Quantification of AKT phosphorylation and CDKN1A expression upon a release from starvation. Means of three experiments + SEM are shown.
(D, E) Immunoblot and quantification of the cMET target c-Myc upon release from DCD. Mean and SEM of three independent experiments are shown, statistical evaluation: paired T test ; * $p<0.05$.

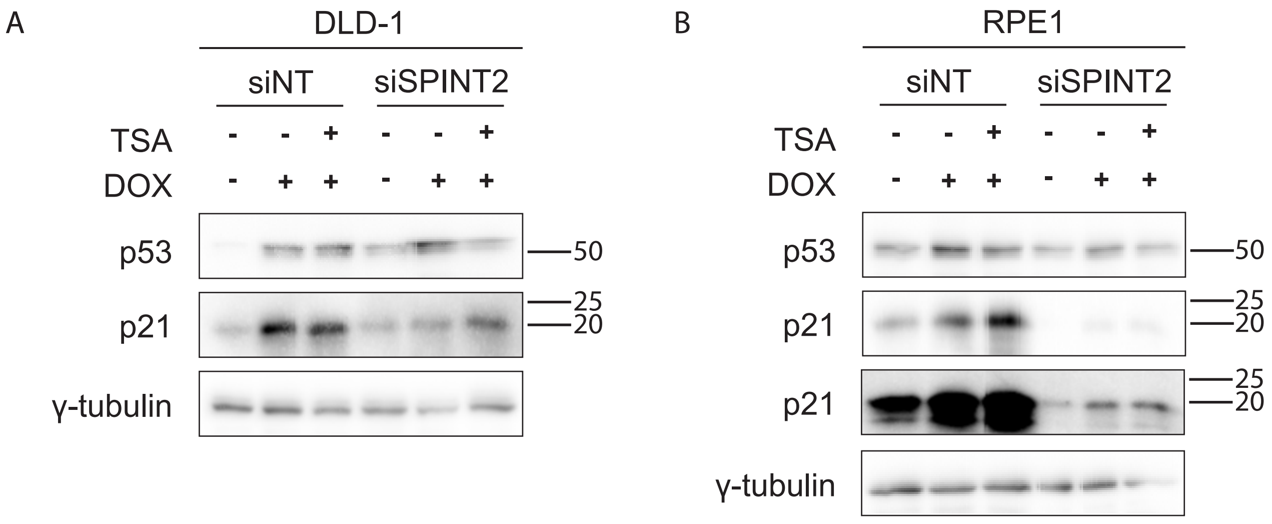

Supplementary Figure 5 Effect of SPINT2 on p21 expression

Representative immunoblot of p53 and p21 activation after TSA and DOX treatment in (A) DLD-1 and (B) RPE1 cell lines.

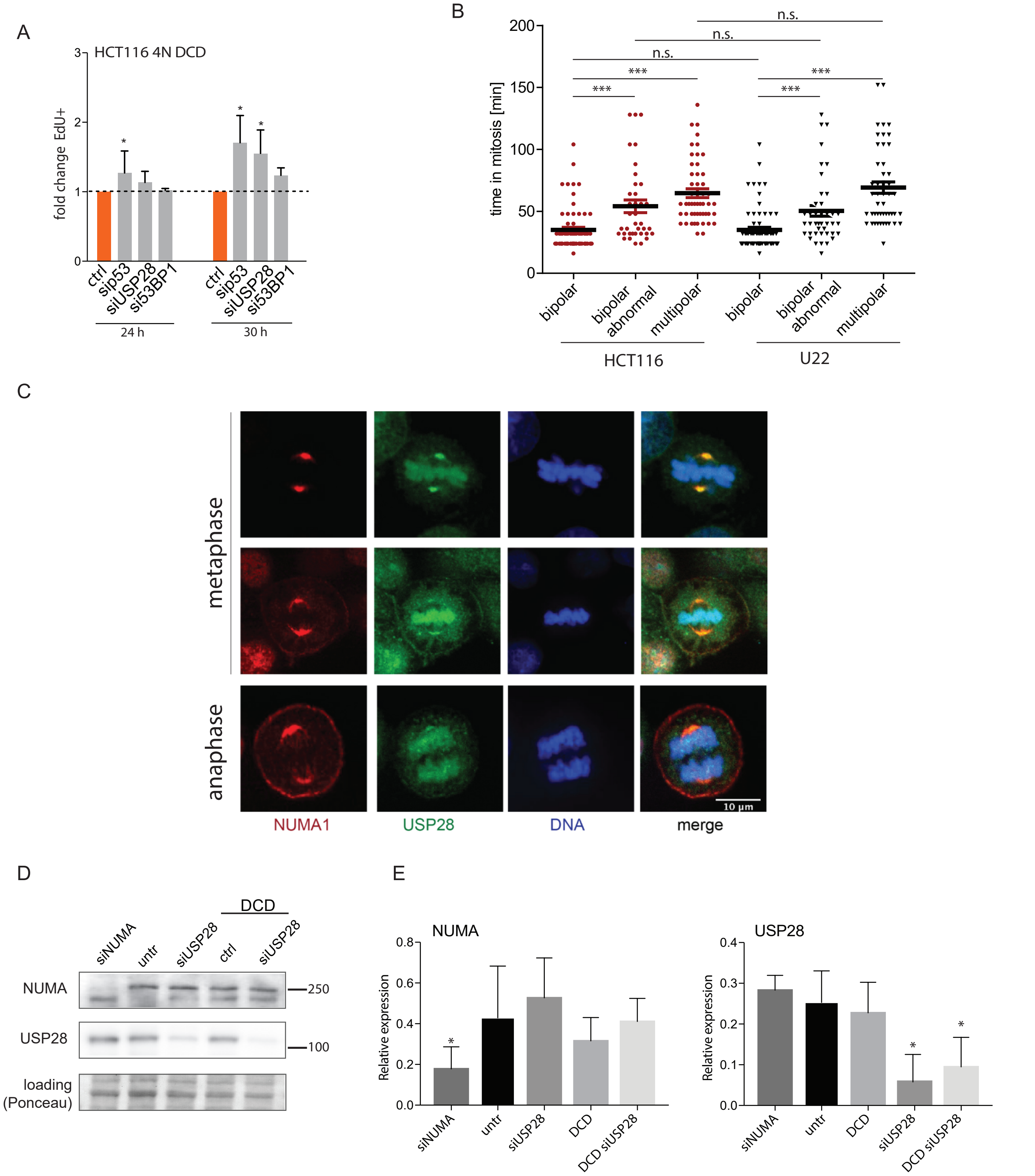

Supplementary Figure 6 USP28 and NUMA1 in response to tetraploidy

(A) Fraction of cells in S phase upon depletion of USP28, p53 and 53BP1. Means of three independent experiments + SEM are shown, normalized to untreated control (ctrl). T test was used for statistical evaluation; * $p< 0.05$; ** $p < 0.01$; *** $p< 0.001$.
(B) Time in mitosis in tetraploid HCT116 and in knock out cell line lacking USP28 (U22). T test was used for statistical evaluation; * $p< 0.05$; ** $p < 0.01$; *** $p< 0.001$.
(C) Localization of NUMA1 and USP28 during mitosis.
(D) Representative immunoblot of NUMA1 and USP28 upon respective depletion.
(E) Quantification of NUMA1 and USP28 levels upon siRNA mediated depletion. Means of three experiments + SEM are shown. T test, * $p<0.05$.

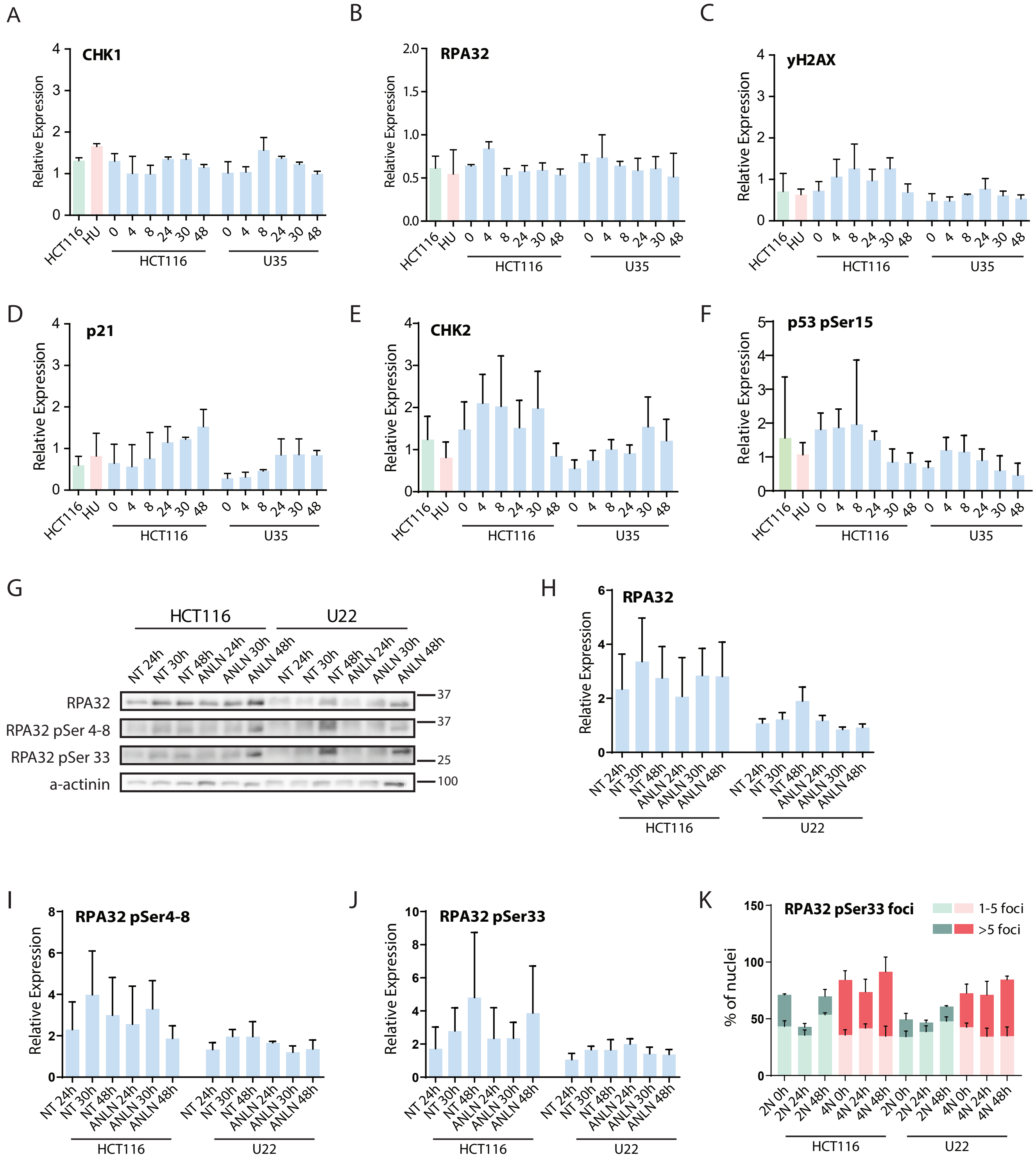

Supplementary Figure 7 DNA damage and checkpoint response in tetraploid HCT116 cells.

(A) Quantification of CHK1, (B) RPA32, (C) yH2AX , (D) p21, (E) CHK2 and (F) p53 pSer15 levels after DCD induced tetraploidization in control HCT116 and in HCT116 cells lacking USP28 (U35).
Means of three experiments + SEM are shown.
(G) Immunoblot of RPA32 and its phosphorylation isophorms after anilin (ANLN) knockdown in wild type HCT116 (left) and after USP28 knockout (U22, right). α-actinin was used as a loading control.
(H) Quantification of RPA32, (I) RPA32 pSer4-8 and (J) RPA32 pSer33 levels after tetraploidization induced by siRNA mediated knockdown of anilin (ANLN) in control HCT116 and in HCT116 cells lacking USP28 (clone U22).
(K) Quantification of RPA32 pSer33 foci in diploid (green) and tetraploid cell populations arising after anillin depletion (red) in HCT116, and in isogenic clones lacking USP28 (U22). Mean + SEM is shown.
